# ‘I think it is helpful … I mean it’s not always helpful’ — diagnostic complexity in endometriosis: a qualitative study

**DOI:** 10.3399/BJGP.2024.0799

**Published:** 2025-11-04

**Authors:** Sharon Dixon, Emma Evans, Katy Vincent, Francine Toye, Abi McNiven, Lisa Hinton

**Affiliations:** 1 Nuffield Department of Primary Care Health Sciences, University of Oxford, Oxford, UK; 2 Nuffield Department of Women’s and Reproductive Health, University of Oxford, Oxford, UK; 3 Physiotherapy Research Unit, Oxford University Hospitals NHS Foundation Trust, Oxford, UK

**Keywords:** diagnosis, endometriosis, general practice, sociological ambivalence

## Abstract

**Background:**

Endometriosis affects approximately 10% of those assigned female at birth. Diagnostic journeys can be complex. The average 8–9 years between presenting symptoms and diagnosis has not changed significantly despite guidance.

**Aim:**

To explore primary care clinicians’ diagnostic considerations in the context of symptoms that suggest possible endometriosis.

**Design and setting:**

Qualitative semi-structured interviews with general practice clinicians working in England.

**Method:**

We report a further analysis of 56 interviews from two inter-linked datasets with GPs and primary care clinicians about supporting patients with symptoms aligned with endometriosis. Analysis was informed by sociologies of diagnosis and ambivalence.

**Results:**

Clinicians valued the importance of diagnoses to patients. Diagnoses support longitudinal care throughout episodes of intermittent specialist input, anticipating and responding to current and future health needs, and delivering evidence-based (biomedical) medicine. Diagnoses help clinicians feel more confident and comfortable, and may confer protection from medicolegal risk. Clinicians balanced these considerations against known uncertainties, including recognition that diagnosis might not change the treatment offered, may not be accessible if empirical trials of treatment relieve symptoms, and that an endometriosis diagnosis may not enable individualised advice or risk prediction. Potential advantages were balanced against diagnostic test risks and system pressures. Recognising that patient care remains with them, GPs anticipate and actively ensure ongoing relationships and care, whatever the outcome of tests. Holding these opposing role- based priorities and expectations in parallel creates tensions, which can be characterised through the concept of sociological ambivalence.

**Conclusion:**

Diagnostic considerations are complex. Educational interventions that do not recognise this may be ineffective in improving or enabling endometriosis diagnostic care journeys.

## How this fits in

Diagnostic journeys for patients with possible endometriosis are often complex, with primary care identified as a pivotal bottleneck in care journeys. It has been suggested that a lack of practitioner awareness about endometriosis contributes to delays in primary care, but research suggests that considerations are more nuanced than simply lacking knowledge. Sociological writing on the meaning of diagnosis offers a framework to consider diagnostic processes and considerations; however, clinician emotions and perspectives are less commonly considered in this work. This article adds to evidence in this space and utilises writing on sociological ambivalence to offer a potential context for clinicians’ diagnostic considerations, with implications for the development of educational interventions.

## Introduction

Although there is some epidemiological uncertainty, endometriosis is thought to affect approximately 10% of people who are assigned female at birth.^1^ It is a heterogeneous condition that can be asymptomatic or contribute to a spectrum of potential symptoms, including pelvic pain, dysmenorrhoea, heavy menstrual bleeding, bladder and bowel symptoms, and fatigue. Endometriosis is associated with possible adverse impacts on fertility, mental health, and quality of life.^1^ The same symptom constellations can also be experienced by women who do not have endometriosis, which makes symptom-based diagnosis challenging.

Many people with endometriosis describe complex journeys to, and through, care. In the UK, there is an average of 8–9 years between presenting potentially relevant symptoms to a health professional and receiving a diagnosis. This may not include time spent living with impactful symptoms before deciding, or feeling able, to seek health care.^2^ The delays in diagnosis have been a focus of health policy, including recent women’s health strategies in England and Scotland.^^2–4^^ The *Women’s Health Strategy for England* ^3^ was informed by a large public consultation; the responses to this echo existing qualitative research in highlighting women’s experiences of not feeling heard when they seek support.^5^ Increased awareness and training, especially among GPs, has been suggested as a strategy to improve diagnostic and care pathways.^3,6^ Despite the development of specific guidance and educational resources for GPs however, diagnostic delays and patient dissatisfaction have not yet demonstrably improved.^2,7^ Knowing how to improve endometriosis diagnosis and care journeys was a top ten uncertainty and research priority identified by the James Lind Alliance.^8^

The 2024 Confidential Enquiry into Endometriosis (conducted by National Confidential Enquiry into Patient Outcome and Death [NCEPOD]) examined care for people with confirmed endometriosis and found that almost 80% of patients presented to GPs before diagnosis, with 58% presenting multiple times before treatment or referral.^9^ Although the enquiry includes examples of excellent holistic care in general practice, 52% of these patients reported not feeling listened to.^9^ The enquiry recommended reframing endometriosis as a chronic long-term condition, with guidance aiming to reduce episodic or fragmented care.^9,10^ The report explicitly considered people who had been diagnosed with endometriosis. Many people investigated for possible endometriosis, with symptoms such as pelvic pain or dysmenorrhoea, do not receive a diagnosis of endometriosis;^11–13^ their care is largely devolved back to general practice,^13,14^ with their needs and experiences largely absent from endometriosis research and considerations.

Our qualitative research, undertaken with GPs in England, explored their considerations when presented with a fictional vignette of a person experiencing symptoms suggesting possible endometriosis. Our research highlighted that, although awareness and knowledge are necessary prerequisites for considering a diagnosis of endometriosis, clinical reasoning is underpinned by complex considerations, including needing to work through a diagnostic hierarchy and the challenges of managing sequential trials of treatment. It also illuminated GPs’ uncertainty about when to refer to secondary care and what best care looked like when empirical trials of treatment were understood to be effective at alleviating symptoms. These complex considerations were contextualised within stretched healthcare systems, including pressure not to refer.^6^

In response to the *Women’s Health Strategy for England*,*^3^* we extended our research to a broader exploration of primary care perspectives on supporting women’s health. This illuminated how primary care practitioners strived to maintain their core commitment to person-centred, life course care in a context of finite resources and complex primary–secondary care interfaces, amid adverse representations of general practice.^14^

Aware of the central focus on, and concerns around access to, endometriosis diagnoses, we were interested in exploring how the concepts and conceptualisation of diagnosis were at work in GP and primary care clinician accounts. To do this, we utilised sociological writing about diagnosis to develop our study.^15,16^ In this article, we expand on this prior research, seeking to identify reflections on diagnosis that represent potential touchpoints for future interventions to improve experiences of accessing and delivering care for symptoms possibly related to endometriosis. Suggestions to improve pathways to endometriosis diagnosis are often predicated on individual-level interventions, such as education and decision aids. However, in conducting our analysis, we were struck by the tensions inherent within clinician reflections about diagnostic considerations, and the structural and health-system contexts these inhabited. We felt that these potentially resonated with Merton’s writing on sociological ambivalence^17^ and utilised this as a theoretical lens to frame our findings.

### Biomedicine and the sociology of diagnosis

Western medicine is underpinned by the biomedical model, which rests upon a number of tacit assumptions, including separating out considerations of mind from body, and a doctrine of specific aetiology, whereby the causes of disease can be identified as biological entities or defined states of dysfunction, which can be identified (and fixed) by doctors. Established critiques of the biomedical model include the falsity of mind–body dualism, the failure of this model to account for sociocultural contexts of disease and illness, and concerns about (over)medicalisation.^18^ Diagnosis and the processes of diagnosis are pivotal tools/concepts in biomedicine, where they substantiate and validate the presence of disease or legitimise illness.

Diagnoses are tools that help classify a broad range of conditions. Jutel’s writing on the sociology of diagnosis shows how a wide range of parameters are used to do this — for example, the presence or absence of a physical structure (cancer, thrombus, congenital anomalies), deviation from statistical norms (hypertension), biochemical anomalies (endocrine disorders), and the presence of constellations of symptoms or experiences (depression).^15,16^ The term ‘diagnosis’ encompasses both the process and endpoint of clinical deliberative reasoning; however, diagnoses are not only endpoints, but also potential entry points to journeys through clinical guidance and evidence-based care. Among other things, diagnoses may enable access to treatment, participation in clinical trials, permission to be ill (social and societal validation), rights to access care (insurance, service pathways), and community membership and advocacy.^19^ Diagnoses and diagnostic processes are dynamic, responding to developing knowledge and societal context, with the power to bestow diagnoses traditionally held by the medical profession.^19^

### Sociological ambivalence

Sociological ambivalence considers social structures and the potentially incompatible normative expectations assigned to particular social roles or status. Whereas psychological ambivalence (or personal uncertainty) is situated within individuals, sociological ambivalence considers how social structures may generate circumstances in which ambivalence becomes embedded.

Sociological ambivalence considers how simultaneous and competing responsibilities can be entrenched within the structure of social statuses, norms, and roles, and recognises the structural factors that create this, along with potential consequences. Merton^17^ proposed that, when competing norms or normative expectations cannot be simultaneously expressed, they may be expressed as behavioural oscillation. He presented these with a series of illustrative couplets, two of which are represented in Box 1,^17^ taken from his essay ‘The ambivalence of physicians’, describing the context of medical education in the US. We utilise and extend this contextual conceptualisation, to reflect on whether this might have resonance as a potential explanatory framing for our findings.

Box 1. **Couplets from Merton’s *Sociological Ambivalence and Other Essays* from the chapter ‘The Ambivalence of Physicians’^17^**

- *‘Physicians should have a strong moral character with abiding commitments to basic moral values.*But: *They must avoid passing moral judgement on patients.’*
- *‘Physicians must provide adequate and unhurried medical care for each patient.*But: *They should not allow any patient to usurp so much of their limited time as to have this be at the expense of other patients.’*

## Method

We undertook a parallel further analysis^20^ of 56 interviews with GPs and primary care clinicians (PCCs) in England: 42 GP interviews about supporting patients with possible endometriosis (2019-2020),^6^ and 14 interviews with primary care clinicians (any clinicians working in general practice settings, including GPs, nurses, advanced nurse practitioners [ANPs], and pharmacists) about supporting women’s health in primary care, including all interviews where the clinician spoke about endometriosis, menstrual pain, or pelvic pain (from a dataset including 46 interviews in total) (2022).^14^ The recruitment strategy and sample descriptions are detailed in Box 2.

Box 2. **Description of sample and participants included in the two studies reported in this analysis**

**Study 1: Navigating possible endometriosis in primary care: a qualitative study of GP perspectives^6^** Sample included 42 GPs (19 male, 23 female) sampled with LCRN support from five regions (Thames Valley and South Midlands, East Midlands, South West Peninsula, Greater Manchester, and North West Coast), enhanced by snowballing. Results have been presented as GP(x), as aligned with numbering in original study.

**Study 2: Understanding primary care perspectives on supporting women’s health needs: a qualitative study^14^** Sample included 11 GPs (10 female, 1 male) and three nurse specialists/ANPs (three female), from a range of locations (including urban and rural settings with an Index of Multiple Deprivation score of 10–90%) and with a range of years in practice (1–25 years). Participants sampled with LCRN support (Yorkshire and Humber, Greater Manchester, Thames Valley, South Midlands, London), enhanced by snowballing. Results have been presented as PCC, as aligned with the numbering in the original study, appended with the participant’s job role (GP or ANP).

ANP = advanced nurse practitioner. LCRN = Local Clinical Research Network. PCC = primary care clinician.

Brief descriptions of both studies are given in Box 2.

Study 2 was commissioned to expand the approach of our original Study 1 and was conducted by the same research team. Both studies utilised comparable approaches to data collection (semi-structured interviews, using clinical vignettes as starting points for clinicians’ reflections on caring for patients with possible endometriosis in general practice), curation, and analysis. Incorporating the additional interviews added reflections from the wider primary care team, as well as accounts of pelvic pain that were outside of the explicit study framing about endometriosis.

We reviewed and re-read in full all of the original study interview transcripts. We included all interviews from Study 1 and all interviews from Study 2 with any mention endometriosis, pelvic pain, or menstrual pain (*n* = 14). We coded and extracted all reflections on, and considerations about, processes and meanings of diagnosis, considerations about decision to undertake investigations or refer for specialist assessment, and on documentation of decisions to refer or investigate and describe the presentation in the GP records. We included all reflections on how any of the above processes would be communicated about or discussed within primary care consultations.

We undertook a thematic analysis using a mind-mapping method (one sheet of paper)^21^ with all authors involved in the development of this further analysis.

Our further analysis research question and coding were iteratively informed by Jutel’s sociology of diagnosis (for example, considering diagnosis as both process and outcome, considering accounts of attributed meanings for GPs and patients).^16^ We also drew on Merton’s theory of sociological ambivalence^17^ to develop and present the conclusion of our analysis.

## Results

We present our findings in a thematic structure that represents clinician reflections on navigating towards a diagnosis of endometriosis. We show how this process is complex and associated with nuanced considerations about diagnostic reasoning and processes. Finally, we show that these considerations and processes are not always emotionally inert for healthcare professionals.

### The value(s) of diagnosis

Conceptually, clinicians regarded disease-based diagnoses as valuable in clinical practice. A major component of this was their recognition of the potential importance and benefits of an endometriosis diagnosis for patients. These included access to specialist care, social and occupational validation of symptoms, membership of support communities, and accessing information and support.

The advantages for patients of having a diagnosis to guide and inform journeys through evidence-based care were also experienced as beneficial for clinicians, enabling them to be more effective at offering evidence-based treatment and managing symptoms. These were conceptualised as shared benefits experienced in clinician–patient relationships and consultations:

*‘*[An] *advantage in the diagnosis would be, you’d be probably more effective at managing their symptoms, you’d probably help them with the psychosocial component of their illness because* [of] *the validation stuff that happens, and also being able to provide targeted treatment appropriately. As opposed to the managing the unknown type symptom, and the advantage also is allowing the patient to make an informed decision.’* GP32

Diagnoses, once attained, could be embedded into a biomedical model of care, where they functioned as a mechanism that could make clinicians’ jobs more straightforward. This included knowing which evidence-based treatments to offer, accessing specialist services and treatments (for example, gonadotrophin-releasing hormone [GnRH] analogue therapy or surgery), informing shared decision making, and anticipating and responding to potential future health needs, including those related to pain or fertility.

As one GP explained, managing future presentations without a diagnosis was akin to working *‘in the dark’* (GP7).

Some GPs reflected that having a diagnosis helped them to feel more *‘confident’* (GP14) or *‘comfortable’* (GP31). Seeking diagnosis was, for some, conceptualised as an intrinsic part of their role as a doctor:

‘[T]*he gut reaction is you, you’ve got to have a diagnosis, but that’s probably just my medical model head. Having been firmly trained for the last* […] *well* thirty *years, so I’d be uncomfortable not having a diagnosis, or at least not even trying to have a diagnosis. I think early stages you’d want to try and have a diagnosis, but you do it, you know, in the expectation that endometriosis is not an easy thing to diagnose without being some, you know, you can get up to the invasive end of the spectrum. Do you really want to do that?* […] *I’m sitting there as a doctor, I want a diagnosis. Because it makes it easier to try and manage, you know it makes my job easier if I’ve got a diagnosis. So, if we know for sure its diagnosis, then we also know that it’s not, you know, anything more sinister.’* GP31

Diagnoses could confer permission to recognise the limits of what GPs could (or should) do, and allow them to seek advice or pass care on to another team. The contribution of protection from potential personal risk, for example, in avoiding complaints or litigation, could also be an important contributory driver towards seeking a diagnosis, although this could also be complex and associated with uncertainty:

*‘… at the end of it, five years down the line saying, “I was worried about my fertility and you did nothing about it.” … then why make it even more stressful and say, “You know, you might be infertile as a result.” And then if they, say, find out it’s not endometriosis, then you’ve got a guaranteed complaint coming your way saying, “You freaked me out unnecessarily.” You know, so. You know I’ve seen it too many times, so no, I’m not going to bring that up.’* GP2

*‘I guess that probably reflects a little bit of my own lack of confidence in the early management of endometriosis. I suppose we want them to get that concrete diagnosis from the specialists, and then you can then sort out the management, but yeah, I’d involve them probably quite early on* […] *And then the medicolegal defensive part of me thinks well, in five years’ time I don’t want a solicitor’s letter pitch up saying, “You delayed referral.”* GP15

Diagnoses could make the clinician’s job *‘nicer’ and ‘easier’* (GP22) because they knew what they were ‘*dealing*’ with (GP7, GP26).

Having a disease-based diagnosis was contrasted against the challenge of managing patients who had undergone specialist assessment and testing, with no resulting unifying diagnosis but with impactful and ongoing symptoms. Chronic pelvic pain was not always conceptualised as a diagnosis, with the implication that an absence of identified pathology represents diagnostic uncertainty, rather than a symptom-based diagnosis of equal parity. Supporting people with pain and no unifying or identified diagnosis could be difficult or challenging; this was potentially compounded when support and management for undiagnosed symptoms fell to primary care clinicians when patients were discharged from secondary care:

*‘Often you’re either not finding anything or what you’re finding isn’t necessarily something that patients are accepting of what you can do about it. I think pelvic pain we do often find quite challenging, and then even when you’ve got the diagnosis, you’re left with the pain, aren’t you, and that can be obviously very difficult to manage, and pain in general’s difficult to manage often.’ PC9, GP*

The drive for a diagnosis meant that patients could *‘kind of fall between people’* (GP30) or *‘end up shopping for* [a] *diagnosis’* (PC18, GP); this could be difficult for both the patient and the clinician:

*‘The expectation from the patient is often quite high, the importance that they place on getting to the bottom of this and getting it fixed is quite high, and together with the mismatch that a large proportion of chronic pelvic pain we don’t ever find a cause for can be really difficult.’* PC8, GP

### Complexities arising when diagnosis (rather than treatment) is an aim of care

Although they valued the role of a disease-based diagnosis, clinicians held a parallel awareness of the need to offer care to those without a diagnosis. Their accounts depict how they work to ensure that patients without diagnoses are not disadvantaged. This reflected the context of general practice, where clinicians continue to hold care and responsibility whatever the outcome of referrals or diagnostic tests.

Clinicians explained how they actively worked to manage patient expectations throughout diagnostic journeys and provide ongoing support, whatever the outcome. The core role of primary care was conceptualised as offering holistic support over time, throughout patient journeys and through healthcare services. This role was, ideally, embedded in ongoing relationships, and characterised by rapport and trust so that the clinician kept the patient *‘onboard’* (PC8 GP), which they actively sought to achieve and sustain:

*‘You know the discussion of yes, you may find an obvious cause and diagnosis for your pain; however, there’s* [a] *significant amount of women with cyclical pelvic pain, but you don’t find an absolute answer for it. It’s about trying to treat the symptoms. So, I’d try and be honest and say, “Yes I agree it would be nice to get a diagnosis, but prepare yourself that we might not.” In which case, we have to think about how we go about, deal*[ing] *with that in terms of symptom control.’* GP39

As well as having uncertain outcomes, clinicians knew that diagnostic and referral pathways took time, describing increasing waiting lists for scans and gynaecology appointments. This meant that, where possible, they wanted to start symptomatic treatment at the same time as referring. While knowing that this might not be possible for fertility concerns, they knew it could be helpful for pain and bleeding.

Whether patients had a diagnosis or not, clinicians recognised that those with ongoing symptoms would likely be returning to primary care for treatment and support. They were aware that patients could rotate around the system, and that this could adversely impact on experiences and diagnostic delays:

*‘Pelvic pain is vague because you obviously explore the menstrual sort of history, sexual history and all sorts, and it doesn’t really sort of yield anything at all, so you end up having to do blood tests and scans, and everything comes back as normal, then you end up referring them on to a secondary care* [specialist] *who say*[s] *they’re not interested because there’s nothing structurally wrong with the scans or there’s nothing wrong in terms of blood, so the patient’s left in limbo, which is quite a difficult thing to sort of manage because the gynaecologist will say, “Well, actually it’s not gynae”*, *but then they won’t suggest what it could be, and you then go down the route of maybe a gastroenterological sort of opinion and they say, “No, it’s not that”, and then the patient’s just left frustrated, and then you feel a bit uncomfortable* [about] *how to manage those.’* PC18, GP

Knowing who to refer, and when, were pervasive concerns balanced against structural limitations, including pressure to not refer and an awareness of over-burdened secondary care services. The sense that *‘can’t refer everyone’* (GP15) could be uncomfortable for clinicians, who had to navigate a precarious balance between over- and underreferral — and therefore, by implication, diagnosis:

*‘There’s a lot of uncertainty and a sort of and, um, you know a lot of difficulty round, um, you know predicting how it’s going to go in the future for patients, um, cos obviously some of them can be referred so then it’s, you know, the specialist manages it, it’s easier in some ways, cos it’s shared isn’t it, the, er, I mean I, myself, I would rather refer everyone because I think it’s quite complicated. But I know with all the, um, you know the pressures of not referring …’* GP34

These broader considerations were amplified by the uncertainties and unknowns about endometriosis as a condition. Clinicians knew that a diagnosis of endometriosis might not change first-line medical management, and that the first-line treatments upon diagnosis are often the same as empirical treatments trialled before referral. This could jar with the perspective that tests should only be undertaken if they would — or likely would — significantly change management. However, clinicians were aware of specialist treatments available, such as GnRH analogues or surgical treatment, which would (or could) not normally be instigated in primary care. This meant that, for patients with ongoing symptoms, referral for these treatments (and/or diagnosis) was clear cut and important.

Clinicians were aware that disease-based diagnoses could facilitate identifying and responding to emerging symptoms, priorities, or concerns. However, they were also aware that there might not be interventions that could reduce or mitigate the likelihood of potential future complications or impacts:

*‘I’m not aware of any of the evidence, but I don’t know whether there* [is] *any prognostic value to diagnosing endometriosis early and being referred for laparoscopic diathermies and things, that they might do to get rid of it.’* GP8

Finally, clinicians reflected that the heterogeneity of endometriosis made it difficult to predict future impacts or needs. Many reflected on the difficulty, even with a confirmed diagnosis, of offering *‘good advice, apart from platitudes in terms of prognosis’* (GP27) because of a lack of evidence about interventions and outcomes. For patients living with chronic pain or seeking referral for difficulty conceiving, the decision to refer for specialist care was clear cut. But, knowing how to advise other patients about potential future impacts, including where endometriosis was identified incidentally, was a real concern. The unpredictability of whether a diagnosis would change treatment or enable effective interventions that could reduce future health impacts, and limitations in being able to meaningfully prognosticate for patients, were balanced against the potential risks of the diagnostic process, notably laparoscopy. This could cause equivocation about the value of diagnosis:

*‘Having a diagnosis of endometriosis is on top of my brain, but what difference is that going to make for her if the treatment I’m doing is helping her with the symptoms? It might give you the satisfaction that, “Okay I, I do know that I have endometriosis”, but if that is being treated whether you’ve been branded with it or without the brand how much I honestly do not know.’* GP41

**Figure 1.**
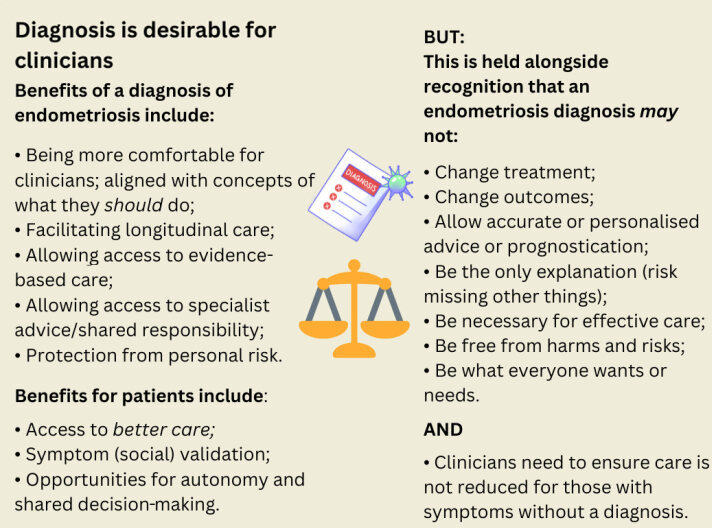
Schematic summary of balanced considerations about endometriosis diagnosis.

The consequence of these uncertainties was that decisions to refer could be complex, comprising potential tensions and uncertainties (Figure 1):

*‘I think what’s difficult, cos you have got to think diagnosis is a thing out there, and it’s a scientific thing, and you can peg everything on it, but it isn’t really. You know it’s a lot more woolly than that. That’s the problem we’ve got.’* GP34

## Discussion

### Summary

Our findings demonstrate primary care clinicians’ complex intersecting considerations around diagnostic processes for possible endometriosis. These include holding the value of having a diagnosis alongside uncertainty about how a diagnosis of endometriosis functions in practice. Considerations were underpinned by wanting to ensure care for all patients, both with and without disease-based diagnoses, and before, during, and after investigations. Our findings suggest a biomedical hierarchy, with precedence given to disease-based diagnoses over symptom-based ones, held alongside an awareness of the limitations of this model in primary care. We demonstrate how primary care clinicians are required to hold potentially competing needs or priorities in parallel and how holding these tensions creates uncertainty. Approaches to managing these tensions included nurturing shared decision-making partnerships with patients.

We highlight clinician accounts of the experience of uncertainty or tensions arising because endometriosis as a condition does not necessarily deliver on the biomedical ideal or promise of how a diagnosis should function in a care pathway. Examples of how an endometriosis diagnosis might not remove uncertainty included whether this helped clinicians know how best to treat, advise on the future, or prognosticate — which are known unknowns in endometriosis care. Diagnoses are pivotal tools within a biomedical framework of medicine, and this creates a driver (magnetic pull) towards diagnosis. We have demonstrated that not being able to achieve or make a diagnosis can be experienced as uncomfortable or associated with disquiet for clinicians. At present, these uncertainties are underpinned by the risks associated with testing for diagnosis (laparoscopy) and awareness of the difficult journeys that patients often experience through services, associated with long waits for care and/or a diagnosis that may not change treatment. Cumulatively, this experiential knowledge — not lack of knowledge — contributed to diagnostic complexity, which is embedded both within clinician role-based expectations and wider societal and health-system constraints.

Taken together, in summary, this is conceptualised in Box 3, where we propose a hypothetical framing developed from our findings, inspired by Merton’s ‘The ambivalence of physicians’.

Box 3. **Diagnostic ambivalence**

- Doctors must make (or strive for) diagnoses (of a disease) as an integral part of care for their patients.*But:* They must not allow the absence of a diagnosis to get in the way of offering care.
- Doctors must offer evidence-based care, often organised around a diagnosis.*But:* They must, in parallel, recognise the limitations of the diagnosis and individualise care, even when they may not be confident that they have the knowledge or resources to do this.
- Doctors must recognise the importance of a diagnosis in validating symptom experiences.*But:* This must happen alongside ensuring that all symptom experiences are valid and validated.
- Doctors must not miss potential diagnoses, and must be aware of the pitfalls of underdiagnosis.*But:* They must be mindful of the risks of medicalisation and potential harms of overdiagnosis.
- Doctors must recognise and respect their patient’s needs and wishes about seeking a diagnosis.*But:* This must sit within rational practice and must take account of the need to not overwhelm services.
- Clinicians must refer individual patients for further or specialist care when clinically appropriate, including balancing (potentially competing) pressures from external guidance and local systems and processes.*But:* They must be aware of service constraints and not overwhelm limited specialist resources.

### Strengths and limitations

This analysis represents a large qualitative dataset comprising 56 interviews with primary care clinicians, conducted over a period of 4 years by the same research team. The team included social scientists, primary and secondary care clinicians (GP, clinical psychologist, gynaecologist). Our analysis was informed by sociological theory.

Further and secondary analysis of qualitative datasets merits careful reflection, acknowledging established critiques about integrating research that was conducted in different contexts or had different original study aims.^22^ However, this extended qualitative analysis was conducted by the same team, using the same methodological approach, and within the same research context (general practice in England). The second dataset was commissioned to mirror the first, and this takes a comparable approach. Study 1 involved only GPs, but Study 2 included a wider range of primary care clinicians. Clinicians in Study 1 were asked explicitly about possible endometriosis; whereas in Study 2 they were asked more broadly about women’s health, with a range of potential scenarios for discussion. Integrating the dataset from Study 2 added value to the analysis, including reflections on managing undiagnosed pain and pain where diagnostic testing has not yielded a unifying explanation for symptoms. This tended to be conceptualised as undiagnosed pain, rather than (diagnosed) chronic pelvic pain syndrome. This added to the range of reflections on meanings of diagnosis. Contextually, this updates impacts of pressure on services, including exacerbation following the COVID-19 pandemic.

Although this qualitative dataset represents a range of clinicians’ roles, experiences, locations, genders, and populations served, we do not suggest that these findings are representative. However, we consider they offer insights into an area of clinical care in which understanding is needed to enable change. Medical professionals’ perspectives on diagnosis are relatively under-represented in sociological writing, and this article contributes to that literature. The framing of our findings within the construct of sociological ambivalence offers an approach to sense making,^23^ but this is proposed as hypothesis only, and as an option for reflection and consideration. It seeks to explicitly and actively situate the challenges clinicians experience inextricably within their roles and health systems. This does not describe individual or personal ambivalence towards individuals or diagnoses, but we recognise the potential for misattribution with this approach. We have endeavored to be clear and transparent in our aims of using theory in this way.

### Comparison with other literature

Experiences of difficult journeys throughout endometriosis care are well documented and remain problematic, despite growing awareness of the condition. Qualitative accounts highlight ways in which people seeking care have found healthcare encounters difficult,^24–26^ including not feeling heard, feeling that their symptoms are trivialised or normalised,^27^ or lacking trust in health professionals’ knowledge about endometriosis, meaning patients *‘battle’* in *‘pursuit of diagnosis’*.^28^

In our studies presented here, clinicians described striving to offer patient-centred, evidence-based, compassionate care, while reflecting on the challenges of doing so. In the original paper published from Study 1, shared decision making, including about uncertainty, emerged as a key finding and recommendation.^6^ Collaborative compassionate care has been identified as a vehicle to improve care experiences in endometriosis.^29^

That unknowns in endometriosis complicate experiences of care is an important contextual factor in our findings.^6^ Reviewing gynaecological journal articles on endometriosis published between 1985 and 2000, Whelan reflects on how the heterogeneity of endometriosis, along with the lack of concordance between symptoms and surgical findings (disease extent), creates uncertainty about the definition of ‘disease’ in endometriosis, creating tensions between science and experience.^30^ This has implications for uncertainty about the meaning of diagnosis, which is, arguably, reflected in the clinicians’ accounts presented here.

One explanation offered to help account for delayed diagnoses in endometriosis is the (mis)attribution of symptoms to other potential causes,^31^ leading to referral to non-gynaecological specialists. This study presented helps explain how this can arise, alongside highlighting clinicians’ frustrations and observations about the impact on their patients. In this study, chronic primary pain diagnoses (for example, chronic pelvic pain syndrome) were not always seemingly recognised as legitimate or helpful alternative diagnoses — instead, symptoms were depicted as unexplained or confusing when occurring in the absence of disease. This is echoed by previous work^13^ and mirrored by patient experiences.^27,32–34^

The endometriosis report commissioned by NCEPOD^9^ calls, as do others, for GPs to have greater awareness and education about endometriosis.^6,25,26,31,35^ Qualitative work with GPs in the Netherlands suggested that they would like this, and offered insights into how this could be shaped by, and for, them — including being embedded in pathways and guidance.^36^ Awareness, guidance, and education about endometriosis is needed, and should help. But, to be effective, resources need to recognise the reality, complexity, and uncertainties of care, including the need to consider differential diagnoses.^6^ There are other conditions whose symptoms can overlap with those of endometriosis, for which delays in diagnosis also have potential adverse impacts on outcomes, including inflammatory bowel disease^37,38^ and gynaecological,^39^ bowel,^40,41^ and urinary tract malignancies.^42,43^ Support in navigating chronic primary pain diagnosis and management is an important need highlighted in this work.

Educational interventions tend to focus on individual, single-event level encounters or interactions, without focusing on systems and the diagnostic journey as a whole. Black *et al* ^44^ proposed a system-level approach when considering how to improve cancer diagnostic journeys, which recognises the general practice reality where people attend with non-specific symptoms that may be misattributed to other conditions. They suggest that taking a systems approach could help patients and clinicians navigate journeys through tests and appointments towards timely diagnosis. We argue that this is potentially also applicable to endometriosis.^45^

Merton^17^ wrote that sociological ambivalence arises when there are incompatible normative expectations assigned to a social status. When these norms cannot be simultaneously expressed, they can become manifested in an oscillation of behaviours, such as between detachment and compassion. Although we could not observe behaviour within our study, we observe oscillatory reflections and considerations about diagnosis, which are perhaps markers for possible behavioural manifestations. We suggest that this framing may offer a lens to help make sense of the slow progress to date in reducing journeys to endometriosis diagnosis, and offer new considerations for interventions developed to mitigate them.

We are not aware of previous work utilising this framing in endometriosis, or seeking to mimic Merton’s paired couplets as a means of reflecting on research findings, but sociocultural ambivalence experienced by clinicians has been described in a number of settings including ‘diagnostic ambivalence’ towards psychiatric diagnostic labelling and the *Diagnostic and Statistical Manual of Mental Disorders* ^46^ in negotiating medication (and medicalisation) in chronic pain,^47^ or in the provision of naloxone prescriptions for patients dependent on opioids.^48^

There are accounts of patient experiences of what is constructed or interpreted as medical ambivalence, including patients with long COVID^49^ or those living with medically unexplained symptoms.^50^ While not explicitly using the sociological construct of ambivalence, in turn, qualitative research exploring junior doctors’ experiences of supporting patients with medically unexplained symptoms highlights contextual challenges and constraints, including concerns about the appropriate use of tests and resources, and balancing medicalisation against risks of ‘missing’ things and organisational constraints;^51^ which resonate with our findings. Qualitative work exploring supporting patients with pelvic pain in primary care also highlights that this may not feel ‘comfortable’ and there is uncertainty about the validity of the diagnostic label.^13^

### Implications for practice

Interventions to improve diagnostic journeys need to account for the reality of the clinical context that, in turn, needs to be considered within wider societal structures.^6^ Our initial study reported that this should include balancing competing diagnostic considerations, represent the whole care journey (navigating through initial tests and trials of treatment, and following up initial and subsequent referrals), meeting the whole-person needs and wishes of the person in the room, and to consider the whole healthcare system (including slow or limited access to specialist care).^6^

This further analysis extends this to argue that these individual-level considerations are embedded within a wider overarching framework of role-based societal expectations and norms, which are sometimes, seemingly, at odds with each other. This can be conceptualised using Merton’s theory of sociological ambivalence. Without adequately accounting for this complexity, we argue that interventions which target individuals predicated only on reductive assumptions that lacking knowledge about endometriosis is the pivotal bottleneck to care might not improve journeys to diagnosis or support. Understanding this could help make sense of why journeys to diagnosis are not changing and inform guidance.

Considerations about diagnosis are not emotionally inert or neutral for clinicians. This analysis adds to the profession’s understanding about complex meanings of diagnosis for primary care clinicians, and may have relevance and resonance in other areas of care and research.
